# Praseodymium-Doped Cr_2_O_3_ Prepared by In Situ Pyrolysis of MIL-101(Cr) for Highly Efficient Catalytic Oxidation of 1,2-Dichloroethane

**DOI:** 10.3390/molecules29143417

**Published:** 2024-07-21

**Authors:** Pengfei Zhu, Zhaoxia Hu, Shouwen Chen

**Affiliations:** School of Biological and Environmental Engineering, Nanjing University of Science & Technology, Nanjing 210094, China; 317102010045@njust.edu.cn (P.Z.); huzhaoxia@njust.edu.cn (Z.H.)

**Keywords:** catalytic oxidation, 1,2-dichloroethane, praseodymium, in situ pyrolysis, synergistic effect

## Abstract

The development of economical catalysts that exhibit both high activity and durability for chlorinated volatile organic compounds (CVOCs) elimination remains a challenge. The oxidizing and acidic sites play a crucial role in the oxidation process of CVOCs; herein, praseodymium (Pr) was introduced into CrOx catalysts via in situ pyrolysis of MIL-101(Cr). With the decomposition of the ligand, a mixed micro-mesoporous structure was formed within the M-Cr catalyst, thereby reducing the contact resistance between catalyst active sites and the 1,2-dichloroethane molecule. Moreover, the synergistic interaction between chromium and praseodymium facilitates O_β_ species and acidic sites, significantly enhancing the low-temperature catalytic performance and durability of the M-PrCr catalyst for 1,2-dichloroethane (1,2-DCE) oxidation. The M-30PrCr catalyst possess enhanced active oxygen sites and acid sites, thereby exhibiting the highest catalytic activity and stability. This study may provide a novel and promising strategy for practical applications in the elimination of 1,2-DCE.

## 1. Introduction

In the past few decades, the emissions of volatile organic compounds (VOCs) have increased dramatically with the rapid development of the industrial economy [1]. Among these compounds, chlorinated volatile organic compounds (CVOCs) have been associated with the generation of photochemical smog pollution and teratogenic effects due to their high chemical stability and severe toxicity [2,3,4,5,6]. One notorious member of the CVOC family is 1,2-dichloroethane (1,2-DCE), which is commonly found in various industries such as paint manufacturing, pharmaceutical chemicals production, and petrochemical operations [7]. Therefore, there is an urgent need to develop cost-effective methods for removing 1,2-DCE. Among the different treatment technologies available, catalytic oxidation is considered a highly efficient and low-temperature operation technology for eliminating CVOCs. However, developing economical catalysts that exhibit both high activity and durability remains a challenge [8]. Firstly, CVOCs present a greater challenge in achieving complete oxidation compared to other VOCs and require higher temperatures for their catalytic degradation [9]. Secondly, the catalyst is susceptible to surface adsorption of chlorine species and carbon deposition, which can potentially lead to its poisoning [10,11]. Therefore, it is crucial to develop exceptionally efficient catalysts that demonstrate superior activity, selectivity (towards CO_2_ and HCl), and stability in order to facilitate practical applications in catalytic oxidation degradation.

Although noble metal (e.g., Pt, Pd, and Ru) catalysts exhibit excellent deep oxidation activity in the destruction of CVOCs, their vulnerability to toxic deactivation, sintering, and high prices have strictly limited practical application [12,13,14,15]. In contrast, transition metal oxide catalysts are more promising and have drawn more attention due to their low cost, superior activity, and stability after modification [16]. Among transition metal catalysts, chromium-based catalysts showed excellent catalytic performance mainly due to the enhanced surface lattice oxygen mobility and strong acidity [17,18,19]. Zhang et al. loaded different transition metals (Cr, Mn, Fe, Ni, and Cu) on a TiO_2_ support for 1,2-DCE oxidation and found that the introduction of Cr showed the best catalytic performance [20]. Separately, rare Earth elements have also received extensive attention due to their unique bonding characteristics and special physical and chemical properties [21]. Particularly, praseodymium oxides showed great catalytic ability owing to their highest oxygen mobility among all undoped rare Earth oxides [21,22,23]. Doping transition metals is an effective strategy to enhance the degradation activity of the catalyst as well as the ability to inhibit chlorine poisoning [16]. It is therefore reasonable to hypothesize that a catalyst combining the advantageous properties of both Cr_2_O_3_ and Pr_6_O_11_ could exhibit exceptional efficiency in the deep degradation of CVOCs. Metal organic frameworks (MOFs) are crystalline materials composed of metal ions or clusters coordinated with organic ligands. MOF materials have been applied in various systems for CVOCs treatment, including air filters, gas masks, and industrial scrubbers. Their high capacity and selectivity suit indoor and outdoor air purification applications. However, MOF materials still lack effective application in high-concentration CVOCs removal.

In this work, aiming to develop highly efficient catalysts for 1,2-DCE oxidation, a series of porous M-PrCrO_x_ catalysts derived from MIL-101(Cr) were prepared with different Pr/Cr molar ratios. Compared with the conventional coprecipitation method, the pyrolysis strategy provides higher surface areas and good diffusion properties [24], resulting in better activity. The synergy effects between Pr and Cr elements in PrCrOx catalysts during 1,2-DCE destruction were investigated by evaluating the physicochemical properties (structural properties, surface composition and element status, reducibility, and surface acidity), catalytic activity, and toxic resistance. It was found that M-PrCr exhibited good catalytic performance and stability, which proved that praseodymium doping and in situ pyrolysis of MOFs are promising strategies to develop effective transition metal oxide catalysts for CVOCs elimination.

## 2. Results and Discussion

### 2.1. Structural Properties

**Figure 1:**
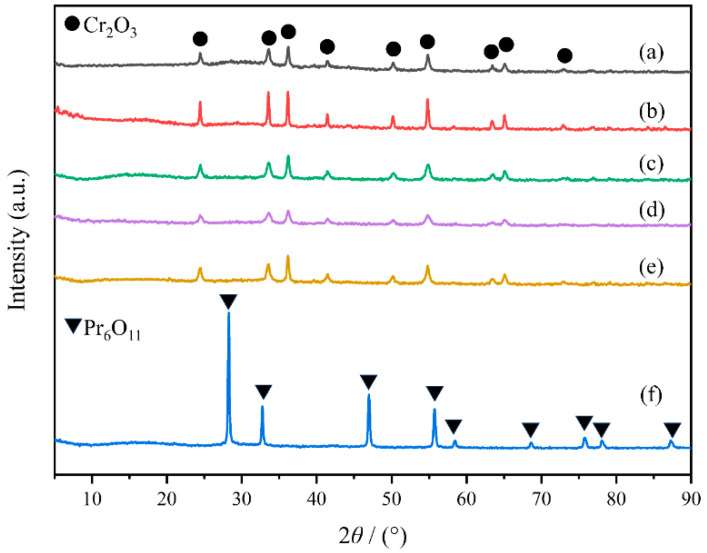
XRD patterns of samples: (a) M-Cr; (b) P-Cr; (c) M-20PrCr; (d) M-30PrCr; (e) M-40PrCr; (f) P-Pr.

As shown in Figure S2, MOF-Cr and MOF-30PrCr have decomposed into M-Cr and M-30PrCr, respectively. Thermogravimetric analysis (TGA) (Figure S1) was applied to further confirm the pyrolysis of MOF-Cr, and the structure of MOF precursor was completely collapsed at around 380 °C, suggesting that the calcination temperature was enough to decompose the MIL-101(Cr) and eventually formed Cr_2_O_3_. The crystal structures of the PrCrO_x_ catalysts were investigated by XRD and the patterns of all samples are presented in Figure 1. Several typical characteristic peaks were observed at 24.49°, 33.60°, 36.20°, 39.75°, 41.48°, 50.22°, 54.85°, 63.45°, and 65.10, as well as 72.94° on the PrCrO_x_ catalysts, corresponding to the (0 1 2), (1 0 4), (1 1 0), (0 0 6), (1 1 3), (0 2 4), (1 1 6), (2 1 4), (3 0 0), and (1 0 10) of the rhombohedral phase Cr_2_O_3_ (PDF#38-1479) [3], respectively. It is evident that all samples displayed similar XRD peaks and showed a good match with the XRD pattern of the standard Cr_2_O_3_ sample, suggesting the successful synthesis of the Cr_2_O_3_ catalyst through MIL-101(Cr) pyrolysis [24]. Separately, P-Pr prepared by precipitation method possessed a cubic phase praseodymium oxide structure (PDF#42-1121) [25]. However, no diffraction peaks of praseodymium species can be observed on the M-PrCr samples, which was attributed to the good dispersion of Pr species in the PrCrO_x_ catalysts. The EDS results also confirmed the Pr element was uniformly dispersed on the M-30PrCr catalysts. According to Scherrer equation, the intensity and width of XRD diffraction peaks are related to the crystal size of the sample [26]. Compared with P-Cr, the diffraction peaks of M-Cr are notably wider, suggesting that pyrolysis strategy provided smaller crystal size of Cr_2_O_3_ [27]. These nano-scaled Cr_2_O_3_ produced more lattice defects and higher surface area of materials [24]. Moreover, the doping of Pr further reduced the crystal size of M-PrCr catalysts, which can provide abundant active sites for the catalytic oxidation. Additionally, P-30PrCr obtained by the co-precipitation method presented very low crystallinity (Figure S2), which might be due to the significant gap in solubility product between Cr(OH)_3_ (Ksp = 6.3 × 10^−31^) and Pr(OH)_3_ (Ksp = 1.5 × 10^−20^). The XRD findings present preliminary evidence that the choice of preparation method significantly influences the characteristics of Cr_2_O_3_ crystal structure, encompassing surface area, lattice defects, crystal size, and other pertinent factors. These factors are crucial for attaining optimal catalytic performance in VOCs oxidation [28,29].

**Figure 2:**
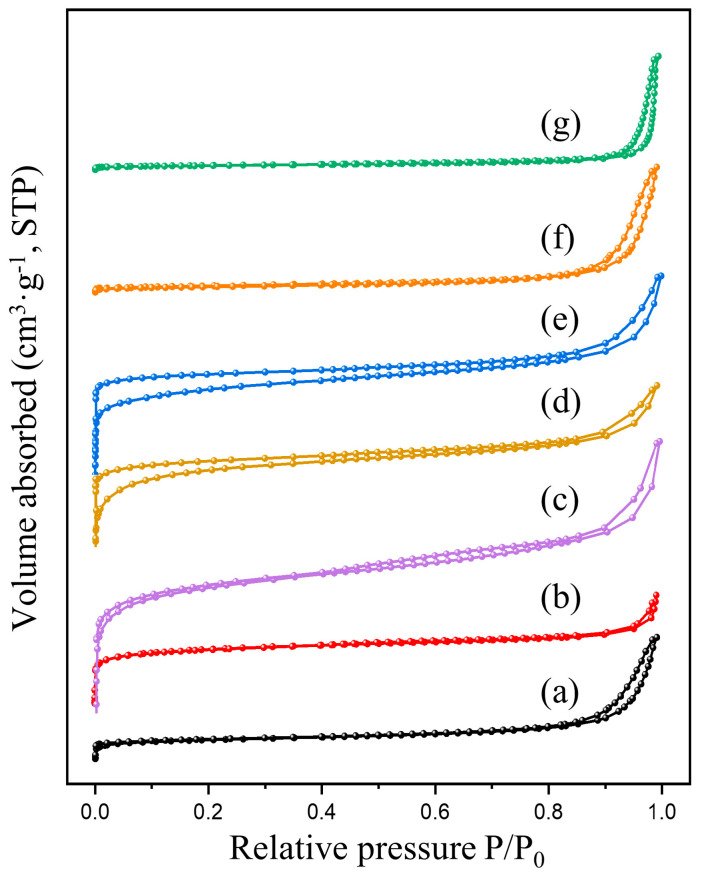
Nitrogen adsorption-desorption isotherms of samples: (a) M-Cr; (b) P-Cr; (c) M-20PrCr; (d) M-30PrCr; (e) M-40PrCr; (f) P-Pr; (g) P-30PrCr.

**Figure 3:**
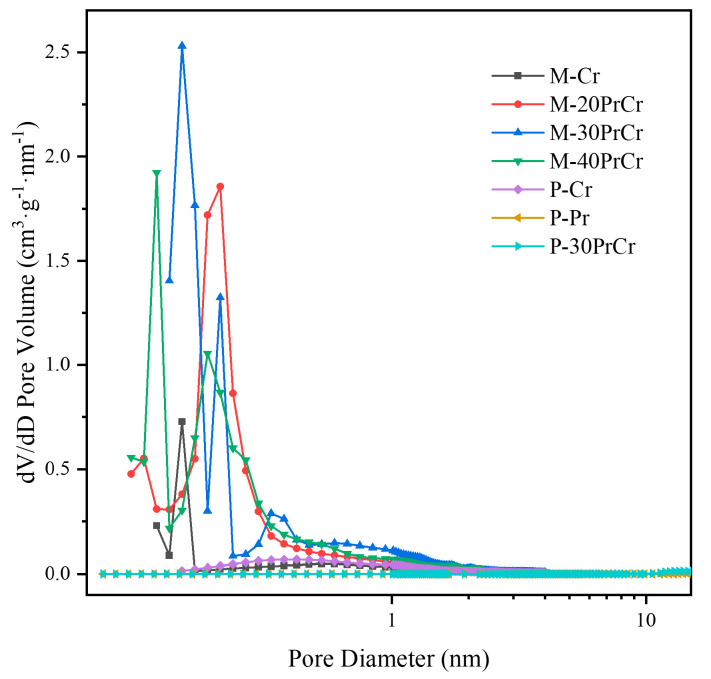
Pore-size distributions of samples prepared via precipitation method and pyrolysis method.

The N_2_ adsorption-desorption isotherms and corresponding pore-size distribution curves for all samples are presented in Figure 2 and Figure 3, respectively. As shown in Figure 3, the prepared catalysts exhibited characteristic IV type adsorption isotherms accompanied by H3 hysteresis loops within the relative pressure range of 0.5–1.0, indicating the presence of a mesoporous structure in the samples. As illustrated in Figure 3, all catalysts synthesized through pyrolysis exhibited a combination of microporous and mesoporous structures, with predominant pore sizes located within the range of 0.5–5 nm. Compared with P-Cr, M-Cr derived from the MOF-Cr precursor exhibited obviously promoted porosity, which improved the diffusion of the 1,2-dichloroethane molecule and reduced the contact resistance between catalyst active sites and 1,2-dichloroethane molecule. With the introducing of praseodymium, the pore size as well as the pore volume of M-PrCr catalysts were gradually enlarged compared with M-Cr catalyst. Separately, the samples synthesized by the precipitation method exhibited bigger irregular mesopore structures and the pore sizes were mainly located at 10–40 nm. The relevant information regarding the BET surface areas, pore volumes, average pore diameters, and catalytic performance of each catalyst can be accessed from Table 1. The decomposition of the ligand led to the formation of numerous irregular pores, resulting in a significant enhancement in the specific surface area (SBET) of M-Cr up to 135.24 m^2^/g. This value is considerably higher than that observed for P-Cr (57.99 m^2^/g). After the introduction of praseodymium (Pr) doping, the specific surface area (SBET value) exhibited a significant increase, with M-30PrCr catalyst demonstrating the highest value at 699.31 m^2^/g. This can be attributed to the moderating effect of praseodymium on the decomposition of terephthalic acid within MOF-Cr, leading to the gradual formation of a more porous structure during pyrolysis. This is further supported by the observed progressive enlargement in average pore size as Pr doping concentration increases on M-PrCr catalysts. After calcination, the formation of PrO_x_ within the CrO_x_ lattice led to the development of novel pore structures, consequently resulting in a gradual augmentation of pore volume. However, the excessive doping of praseodymium blocked certain pore structures, resulting in a reduction in both specific surface area and pore volume. Separately, the coprecipitation method yielded only a slight improvement in specific surface area and pore volume for P-30PrCr compared to P-Cr. It is widely acknowledged that an increased pore volume and higher specific surface area can have beneficial effects on catalytic activity when it comes to oxidizing volatile organic compounds [30].

**Figure 4:**
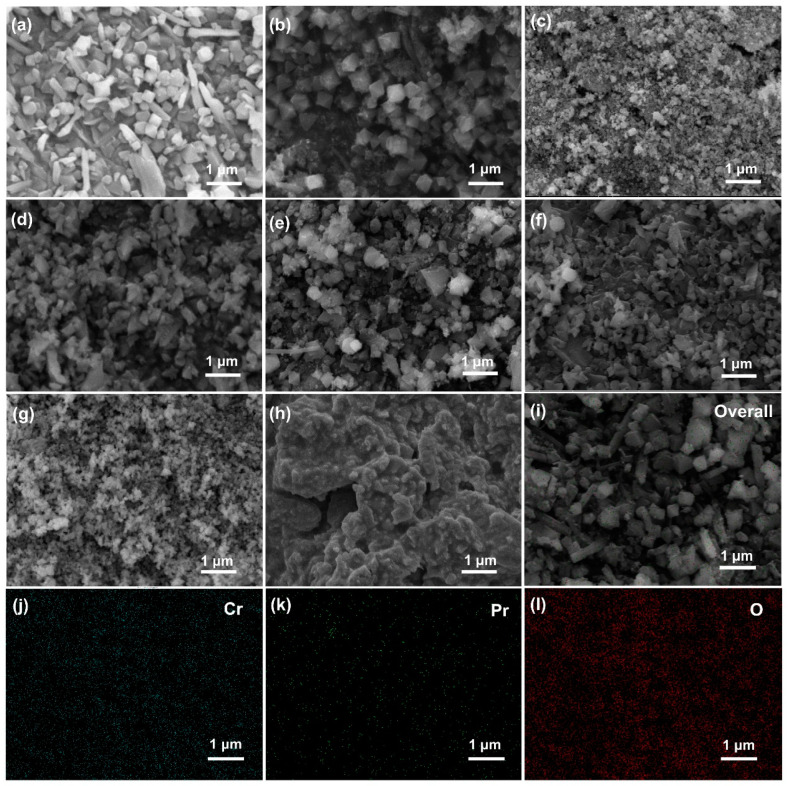
SEM images of the PrCr catalysts with different Pr/Cr ratios (**a**–**h**): (**a**) MIL-101(Cr), (**b**) M-Cr, (**c**) P-Cr, (**d**) M-20PrCr, (**e**) M-30PrCr, (**f**) M-40PrCr, (**g**) P-Pr, (**h**) P-30PrCr, and EDS mapping result for M-30PrCr: (**i**) selected section, (**j**) Cr (Lα1), (**k**) Pr (Mα), and (**l**) O (Kα1).

The catalyst structure is vital to catalytic performance. Hence, morphology and element distribution of the catalysts were investigated by SEM and EDS mapping tests. As shown in Figure 4c,g, catalysts prepared with precipitation method displayed irregular granule structure with porosity and looseness. However, small particles of P-Cr were agglomerated to large plates after Pr doping, which blocked the pore structure and reduced the specific area of the catalyst. As shown in Figure 4a, MIL-101(Cr) precursor with octahedral structure was prepared by fluorine-free hydrothermal method. After being calcined at 400 °C for 4 h, the smooth surface of MIL-101(Cr) became rather rough, which is due to the terephthalic acid of MIL-101(Cr) precursor being pyrolyzed, leaving octahedral porous Cr_2_O_3_ nanosatellites being noted as M-Cr. With the doping of Pr, the octahedral structure of M-20PrCr and M-30PrCr was partially altered, presenting smaller particle sizes and higher specific surface area. The highest specific surface area was found in M-30PrCr; the partially damaged octahedral structure enhanced the specific area, which promoted contact with CVOCs, thus benefiting the catalytic performance. Additionally, the excessive doping of Pr hindered the formation of MOF precursors, resulting in the irregular blocky structure of M-40PrCr, which significantly reduced the specific area of the catalyst. The elimination of CVOCs can be influenced by the specific area of the catalyst, as it has the potential to affect the accessibility of catalytic active sites. These findings are consistent with the results obtained from XRD and N_2_ adsorption/desorption analyses.

As shown in Figure 4i–l, the surface element distribution of the M-30PrCr catalyst was determined through EDS analysis. Randomly selected regions were scanned and subjected to EDS analysis, revealing a uniform dispersion of Cr, Pr, and O elements on the catalyst surface. This can be attributed to the presence of an amorphous crystal structure observed in the XRD patterns. Additionally, the molar ratio of Pr/Cr (0.096) obtained from EDS analysis was found to be significantly lower than the theoretical value (0.30). This observation suggests that Pr has been incorporated into the lattice of CrO_x_, facilitating interaction between Pr and Cr. Consequently, it is inferred that doping with PrO_x_ may hinder the crystallization process of CrO_x_. Ultimately, by employing pyrolysis method along with appropriate proportions of Pr doping, modifications can be made to both structure and morphology of catalysts leading to developed catalytic activity. Notably, among all tested catalysts, M-30PrCr demonstrated superior performance in this regard.

### 2.2. Surface Composition and Element Status

**Figure 5:**
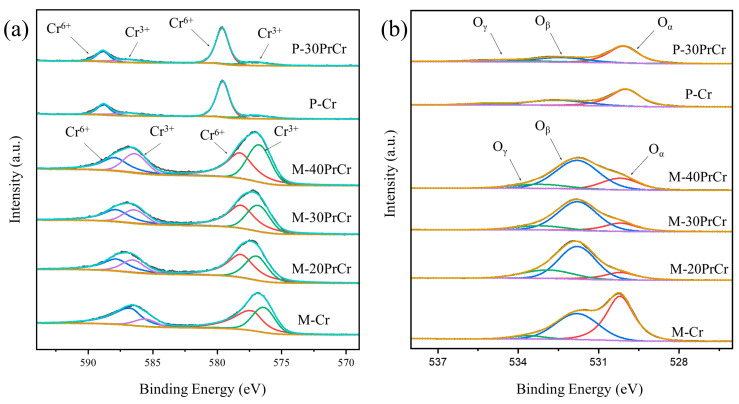
The XPS spectra of the catalysts: (**a**) Cr 2p; (**b**) O 1s.

The surface elemental compositions, chemical valence states, and oxygen species of the prepared catalysts were analyzed using XPS technique. Corresponding results are presented in Figure 5, which displays the XPS spectra for Cr 2p, O 1s, and Pr 3d signals.

Figure 5b shows the spectra of O 1s, which is decomposed into three peaks located at 530.2, 531.8, and 533.2 eV. Among them, the peak observed at about 530.2 eV corresponds to lattice oxygen (O_α_.), primarily originating from Cr and Pr. The peaks located at around 531.8 eV can be attributed to the presence of surface-adsorbed oxygen, indicating the existence of O_β_ species. Meanwhile, the peak detected at 533.0–533.4 eV corresponds to carbonates and chemisorbed water (O_γ_) on the catalyst’s surface [18,31,32]. The O_β_/(O_α_ + O_β_) ratio was determined by calculating the areas of Oα and O_β_, as presented in Table 2. The prepared catalysts exhibited the following order of O_β_/(O_α_ + O_β_) ratios: M-30PrCr (0.81) > M-40PrCr (0.79) > M-20PrCr (0.74) > P-Cr (0.45) > M-Cr (0.32) > P-30PrCr (0.30). The introduction of Pr alters the valence state of Cr, leading to a significant increase in the proportion of O_β_. In general, O_β_ demonstrates higher activity compared to O_α_ due to its relatively enhanced mobility and reduced surface bonding energy [33]. The high concentration of O_β_ in M-30PrCr contributes to its exceptional catalytic activity at low temperatures.

The Pr 3d spectra of M-PrCr and P-PrCr catalysts are decomposed into five peaks, denoted as v and u, corresponding to the energy levels of Pr 3d3/2 and Pr 3d5/2, respectively. As depicted in Figure S5, the peaks v′ (933.1 eV) and u′ (928.4 eV) are attributed to surface Pr^3+^ species while the remaining peaks are assigned to surface Pr^4+^ species [34,35,36,37,38]. The results demonstrate the presence of Pr^3+^ and Pr^4+^ in a mixed valence state on the P-30PrCr surface, indicating the formation of oxygen vacancies [39]. However, due to the significant dispersion of Pr, the characteristic peak associated with Pr cannot be observed on the surface of the M-PrCr catalyst.

As shown in Figure 5a, The Cr 2p spectra of the catalysts are resolved into four peaks. The Cr 2p_3/2_ bond energies of Cr^3+^ and Cr^6+^ are approximately located at 576.4 eV and 578.7 eV, and the bands located at 586.2 eV and 588.2 eV are attributed to the Cr^3+^ and Cr^6+^ of the Cr 2p_1/2_ spectra, respectively [31,40,41], revealing the coexistence of Cr^3+^ and Cr^6+^ on the catalyst surface. The ratios of Cr^6+^/Cr^3+^ are 0.97, 2.16, 1.23, 1.48, 1.41, and 4.07 for M-Cr, P-Cr, M-20PrCr, M-30PrCr, M-40PrCr, and P-30PrCr, respectively. With the addition of Pr, the proportion of Cr^6+^ initially increases and then decreases. The appropriate doping of Pr can effectively enhance the content of Cr^6+^. The results presented above demonstrate that the interaction between Pr_6_O_11_ and Cr_2_O_3_ enhances the presence of Cr^6+^ and O_Latt_ on the catalyst surface, which collectively contribute to an improved catalytic performance in 1,2-DCE combustion. Based on the XPS analysis findings, it can be concluded that M-30PrCr exhibits superior catalytic activity for 1,2-DCE combustion, aligning with the conclusions drawn from catalytic performance evaluation in Section 3.4. 

### 2.3. Surface Redox Properties and Acid Properties

**Figure 6:**
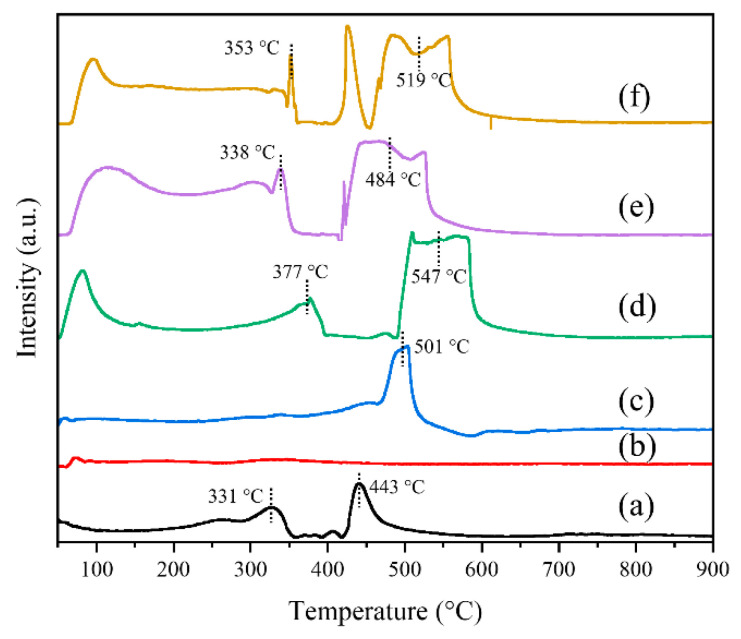
H_2_-TPR curves of praseodymium chromium composite oxides catalysts: (a) M-Cr, (b) P-Cr, (**c**) P-Pr, (d) M-20PrCr, (e) M-30PrCr, (f) M-40PrCr.

The reducibility at low temperatures is a crucial factor in determining the catalytic efficiency of metal oxide catalysts [18]. To evaluate the redox property of the PrCrO_x_ catalysts, H_2_-TPR technique was conducted over a temperature range spanning from 50 to 900 °C. The reduction peaks in the ranges of <250 °C, 250–400 °C and 400–650 °C could be assigned to the reduction of physically absorbed oxygen, surface chemical oxygen, and lattice oxygen species [42,43]. As shown in Figure 6, the reduction peak of P-Pr appeared at 501 °C, which represents the reduction of the surface Pr_6_O_11_ [35,44]. Compared to P-Cr catalyst, the M-Cr exhibited significantly enhanced reducibility. The peaks observed at 331 °C and 443 °C were attributed to the reduction of Cr^6+^ to Cr^3+^ and the subsequent reduction of Cr^3+^ to metallic Cr, respectively [18,32]. Compared with M-Cr, the reduction peak of M-PrCr catalyst is significantly enhanced, indicating that the introduction of Pr has greatly developed the reducibility of the catalyst. Furthermore, with an increase in the doping amount of praseodymium, there is a noticeable shift of the reduction peaks towards a lower temperature range. This observation suggests that the introduction of an optimal concentration of Pr significantly enhances the mobility of oxygen species. The quantification of H_2_ consumption was conducted to evaluate the reducibility of catalysts at temperatures lower than 700 °C, as indicated in Table 2. The amount of H_2_ consumption (<700 °C) exhibited a descending trend as follows: M-40PrCr (28.37 mmol⋅g^−1^) > M-30PrCr (27.24 mmol⋅g^−1^) > P-Pr (14.19 mmol⋅g^−1^) > M-20PrCr (10.61 mmol⋅g^−1^) > M-Cr (7.10 mmol⋅g^−1^)> P-Cr (5.79 mmol⋅g^−1^), demonstrating that M-40PrCr exhibited the highest amount of H_2_ consumption. These findings suggest that the M-30PrCr catalyst exhibits improved reducibility, facilitating the efficient transport of oxygen species and initiation of reactants [45].

**Figure 7:**
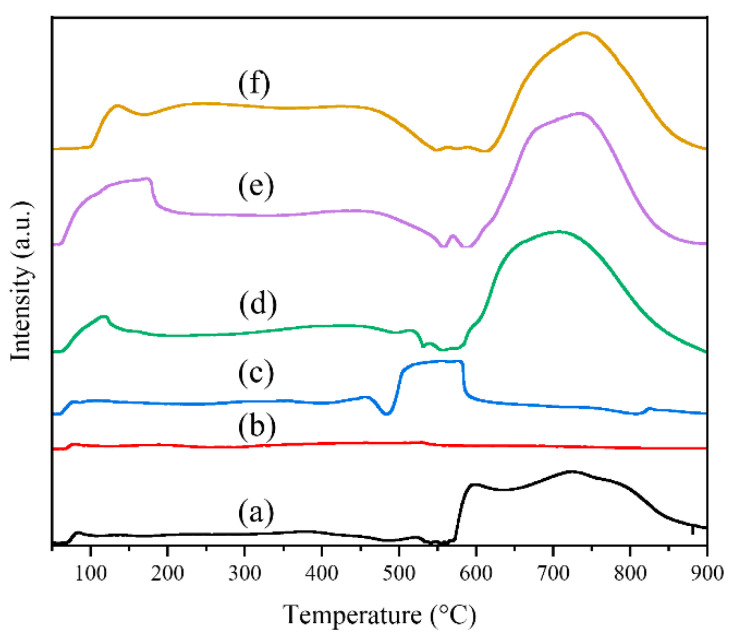
NH_3_-TPD curves of praseodymium chromium composite oxides catalysts: (a) M-Cr, (b) P-Cr, (c) P-Pr, (d) M-20PrCr, (e) M-30PrCr, (f) M-40PrCr.

The NH_3_-TPD technique was utilized to assess the acidity of the prepared catalysts, and Figure 7 illustrates the corresponding results. Desorption peaks corresponding to NH_3_ absorption on acid sites categorized as weak, medium, and strong are observed at temperatures below 300 °C, between 300–600 °C, and above 600 °C respectively [20,31,33]. In general, it is widely acknowledged that deep oxidation efficiency for CVOCs is higher on strong acid sites compared to weak acid sites [40]. The quantities of acid sites were calculated and the results are presented in Table 2, and the order of medium and strong acid sites is as follows: M-40PrCr (763) > M-30PrCr (722) > M-20PrCr (697) > M-Cr (578) > P-Pr (251) > P-Cr (74). The acidity of the M-Cr catalyst, prepared by pyrolysis method, is significantly stronger than that of P-Cr catalyst, prepared by co-precipitation method. Furthermore, the introduction of praseodymium doping resulted in a shift towards higher temperatures for desorption peaks, indicating a significant enhancement in the acidity of M-PrCr catalysts. It is widely recognized that the presence of strong acid sites enhances the dissociative adsorption of chlorine from CVOCs [18,32,45]. Apparently, the incorporation of praseodymium species significantly enhances the abundance of potent acidic sites in PrCrO_x_ catalysts, thereby facilitating the release of chlorine from 1,2-DCE [46].

### 2.4. Catalytic Performance and Structure-Activity Relationship

**Figure 8:**
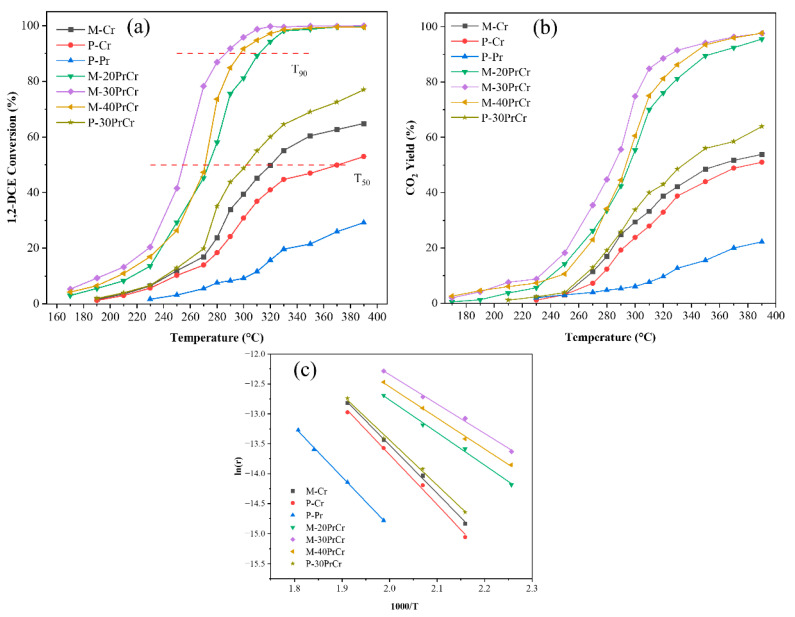
The catalytic performance for 1,2-DCE oxidation over the prepared catalysts: (**a**) Conversion rate, (**b**) Complete oxidation rate, (**c**) Apparent activation energy.

Catalysts with varying molar ratios of Pr/Cr were utilized for the catalytic oxidation of 1,2-dichloroethane. The relationship between temperature and Pr/Cr on 1,2-dichloroethane conversion is presented in Figure 8a. In general, the conversion efficiency of 1,2-DCE increased with the temperature rise and presented a S-shaped curve, suggesting that high temperature is conducive to 1,2-DCE elimination. The T_50_ (the temperature of 50% 1,2-DCE conversion) of P-Cr, M-Cr, P-30PrCr, M-20PrCr, M-40PrCr and M-30PrCr catalysts is 371 °C, 320 °C, 302 °C, 274 °C, 271 °C and 255 °C, respectively. The corresponding T_90_ (the temperature of 90% 1,2-DCE conversion) for M-20PrCr, M-40PrCr and M-30PrCr is 312 °C, 298 °C and 286 °C, respectively. The T_50_ of P-Pr was above 400 °C, indicating that Cr_2_O_3_ provided the main active sites. It is obviously seen that P-30PrCr presented lower T_50_ than pure P-Cr and P-Pr, which indicates that the interaction between Cr_2_O_3_ and Pr_6_O_11_ can improve the catalytic ability for 1,2-dichloroethane oxidation. Notably, M-Cr derived from MIL-101(Cr) exhibited higher catalytic performance than P-Cr, indicating the porous structure generated by pyrolysis method promoted the contact between CVOCs and catalyst. Moreover, the catalytic performance of M-PrCr catalysts (M-20PrCr, M-30PrCr and M-40PrCr) are higher than M-Cr and P-30PrCr in 1,2-DCE oxidation, and M-30PrCr presented the highest activity. These results could conclude that the doping of Pr significantly promoted the activity of the M-PrCr catalysts for 1,2-DCE oxidation. This might be attributing to the synergistic effect between chromium and praseodymium, causing the distortion of chromium lattice and the improved oxidation performance of 1,2-DCE.

Due to their exceptional chemical stability, CVOCs are difficult to completely oxidize, leading to the formation of highly toxic intermediates such as dioxins [8]. Therefore, in practical applications, complete mineralization is even more important than a lower conversion temperature. As demonstrated in Figure 8b, the mineralization rates exhibit a similar trend to the conversion rates observed on the catalyst. It is shown that the M-30PrCr catalyst doped with Pr presented excellent oxidation ability, and complete oxidation of 1,2-DCE was achieved at about 360 °C. Compared to the co-precipitation method, the pyrolysis method offers a higher abundance of low-temperature reduction sites for CrO_x_, enhances the presence of active oxygen species on the catalyst surface, thus benefits the complete oxidation of 1,2-DCE. Moreover, the introduction of Pr generated new oxygen vacancies and significantly improved the acidity of the catalysts, thereby further facilitated the elimination of 1,2-DCE.

To further investigate the reaction mechanism, the Arrhenius plots with linear fitting was presented in Figure 8c. From these plots, the apparent activation energy (E_a_) for the oxidation of 1,2-DCE was determined using the Arrhenius equation [47]. The order trend of calculated E_a_ is P-Pr (74.9 kJ/mol) > P-Cr (69.4 kJ/mol) > M-Cr (65.9 kJ/mol) > P-30PrCr (62.5 kJ/mol) > M-20PrCr (45.2 kJ/mol) > M-40PrCr (43.1 kJ/mol) > M-30PrCr (40.8 kJ/mol). Clearly, praseodymium-doped catalysts presented lower activation energy than pristine P-Cr, which was agreeing well with the results of activity tests. Compared with P-30PrCr, M-30PrCr prepared by pyrolysis method presented lower activation energy, indicating that the pyrolysis method facilitated the interaction between chromium and praseodymium. These results confirmed the pyrolysis method and the introduction of praseodymium species significantly enhanced the oxidation ability of CrO_x_ catalysts.

### 2.5. Durability Test

**Figure 9:**
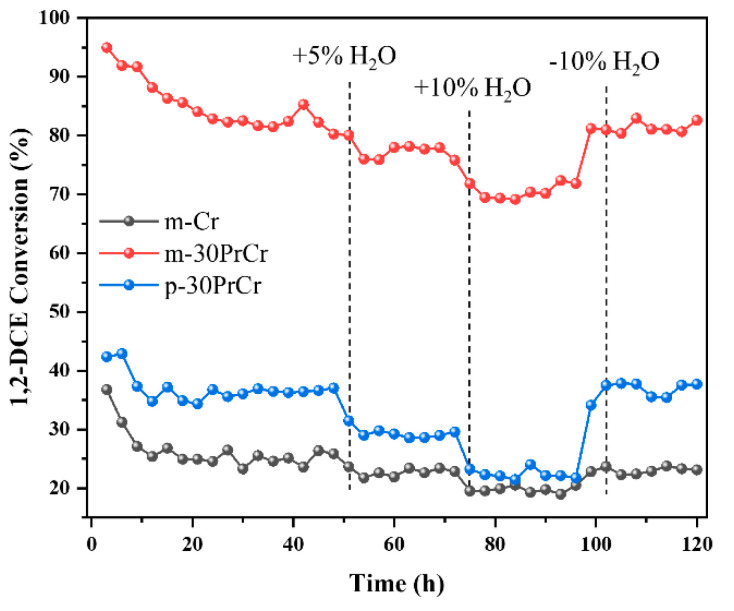
Durability test for catalytic oxidation of 1,2-DCE over PrCrOx catalysts at 290 °C.

To investigate the durability of PrCrO_x_ catalysts, an on-stream reaction experiment was carried out at 290 °C. As shown in Figure 9, M-30PrCr exhibited remarkable durability for 1,2-DCE oxidation over a prolonged period of 100 h. In the initial 48-h period, the conversion of 1,2-DCE over M-30PrCr decreased from 95% to 82%, attributed to the accumulation of chlorine species and carbon. Separately, the conversion over M-Cr decreased from 44% to 36% and the conversion over P-PrCr decreased from 37% to 26%. Additionally, the influence of H_2_O on the catalytic activity was also studied. After the introduction of 5 vol.% water vaper, the conversion of 1,2-DCE over M-30PrCr gradually dropped to 78%, followed by a further decrease to 70% with the introduction of 10 vol.% water vapor. A similar situation was observed on the M-Cr catalyst, where the conversion decreased from 23% to 20% with the increase of water vapor. The observed phenomenon can be attributed to the competitive adsorption of water on the active sites of the catalysts. Interestingly, when H_2_O is removed, the conversion of 1,2-DCE over M-30PrCr and P-30PrCr was recovered to a higher level than that after 48h. This might be due to the fact that the presence of water vapor developed hydroxyl groups on the catalyst surface, facilitating the desorption of Cl species and thereby mitigating catalyst deactivation [48]. However, only partial conversion over pristine M-Cr was recovered after the removal of water vapor, indicating pristine Cr_2_O_3_ is vulnerable to Cl poisoning. The durability test results show that Pr doping can effectively enhance the Cl resistance of CrO_x_ catalysts, among which M-30PrCr has excellent durability against the effects of 1,2-DCE and H_2_O.

### 2.6. Characterization of Used Catalysts

**Figure 10:**
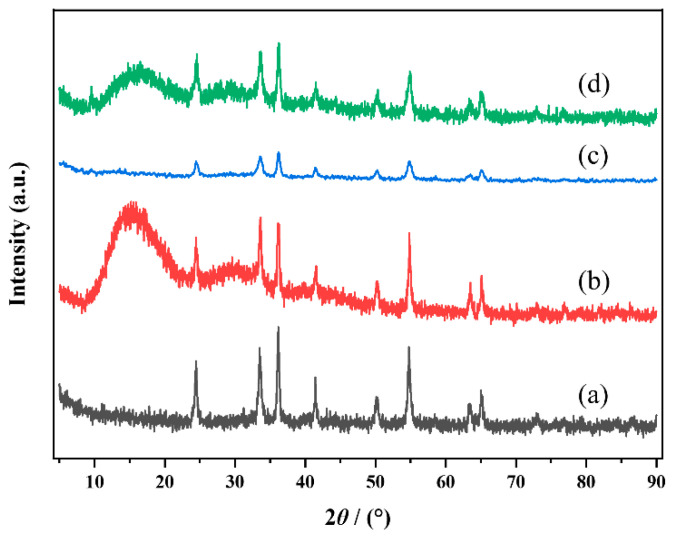
XRD patterns of used catalysts: (a) M-Cr; (b) used M-Cr; (c) M-30PrCr; (d) used M-30PrCr.

**Figure 11:**
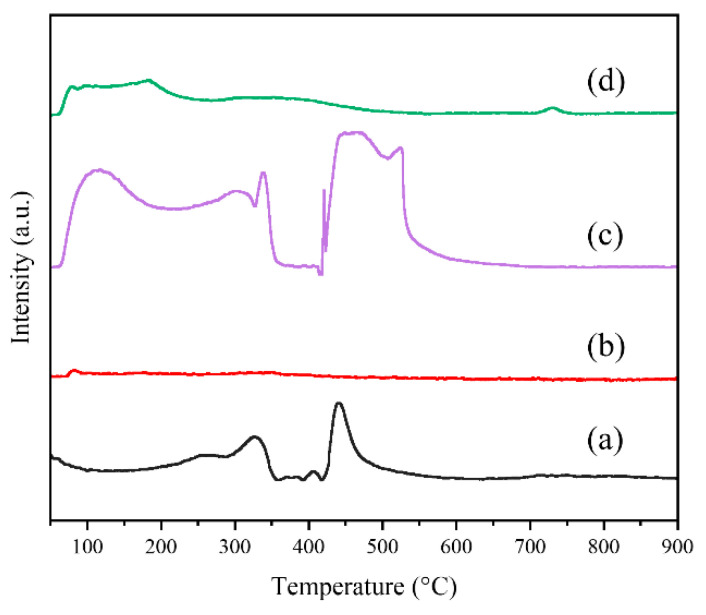
H_2_-TPR curves of used catalysts: (a) M-Cr, (b) used M-Cr, (c) M-30PrCr, (d) used M-30PrCr.

To gain further insights into the reaction behavior of the PrCrO_x_ catalysts during 1,2-DCE degradation, a comprehensive analysis including XRD, SEM, EDS and H_2_-TPR was conducted on the used catalysts. As shown in Figure 10, the X-ray diffraction patterns of both M-Cr and M-30PrCr exhibit the appearance of distinct broad peaks at an angle of 2θ = 16°. This phenomenon suggests that carbon species were accumulating on the catalyst surface during the durability assessment. It is obvious that the new peak of used M-30PrCr is much weaker than that of used M-Cr, indicating that the presence of Pr can effectively inhibit carbon deposition. Separately, the characteristic peaks of the used catalysts were enhanced compared with that of the fresh catalysts, which may be due to the sintering of the catalyst. Figures S3 and S4 depict the SEM and EDS mapping images, respectively, while Table S1 presents the corresponding results. After the durability tests, the deposition of C and Cl species was found on the surface of both M-Cr and M-30PrCr catalysts. However, compared with M-Cr, the accumulation of C and Cl species on the surface of M-30PrCr catalyst was significantly reduced, indicating that Pr doping can inhibit catalyst poisoning. The H_2_-TPR curves are presented in Figure 11. After the durability test, the reduction peaks located in the high-temperature area almost completely disappeared, and the reduction peaks in the low-temperature area also weakened significantly. This phenomenon illustrates that the surface chemical oxygen of the catalysts played a crucial role in CVOCs oxidation. Compared with M-Cr, the reduction peaks of M-Pr are better preserved, indicating that the introduction of Pr is helpful for the migration of oxygen species, thus enhancing the lifetime of the catalyst.

## 3. Materials and Methods

### 3.1. Chemicals and Materials

The praseodymium nitrate hexahydrate (Pr(NO_3_)_3_·6H_2_O), chromic nitrate nonahydrate (Cr(NO_3_)_3_·9H_2_O), and terephthalic acid (H_2_BDC) were purchased from Aladdin Biochemical Technology Co., Ltd., Shanghai, China. The 1,2-dichloroethane (DCE), ethyl alcohol, nitric acid, and sodium hydroxide were purchased from Sinopharm Chemical Reagent Co., Ltd., Shanghai, China. All reagents were analytical grade and used without further purification.

### 3.2. Catalyst Preparation

#### 3.2.1. Synthesis of MIL-101(Cr)

The synthesis of MIL-101(Cr) was achieved using a hydrothermal method without the use of fluorine [49]. In this procedure, 4 g of Cr(NO_3_)_3_·9H_2_O and 1.66 g of terephthalic acid were dissolved in 48 mL of deionized water and stirred at room temperature for 1 h. Subsequently, a modulator consisting of approximately 1 mL nitric acid (~60%) was added to the solution, followed by ultrasonication for 1 h. The reaction took place in a Teflon-lined autoclave at a temperature of 210 °C for a duration of 8 h. After cooling, the resulting product was separated through centrifugation and subjected to heating in ethanol (100 mL) for an extended period of time (24 h). Finally, the obtained green powder was collected via centrifugation and dried at a temperature of 80 °C over a period lasting 12 h.

#### 3.2.2. Synthesis of M-Cr and M-xPrCr

**Figure 12:**
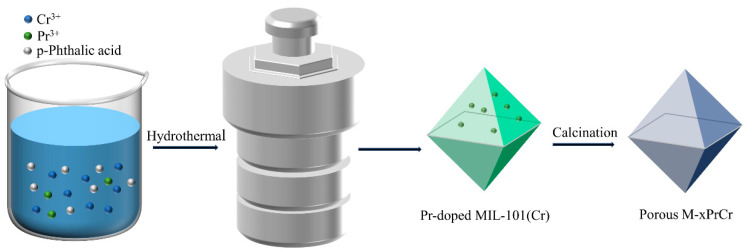
Synthetic process of M-xPrCr catalysts via in situ pyrolysis method.

As shown in Figure 12, the M-Cr and M-xPrCr catalysts were synthesized via in situ pyrolysis of the MOF-Cr precursor. Typically, the sacrificial precursor MIL-101(Cr) was calcined at 400 °C under an air atmosphere for 4 h with a heating rate of 5 °C·min^−1^. The obtained porous Cr_2_O_3_ was marked as M-Cr. Praseodymium-doped Cr_2_O_3_ was prepared with a similar method. Typically, a certain mass of Pr(NO_3_)_3_·6H_2_O (theoretical addition amounts are determined by the atom molar ratio of Pr/Cr, e.g., M-20PrCr requires about 0.87 g of Pr(NO_3_)_3_·6H_2_O), which was dissolved into the precursor solution, and other conditions were the same as the synthesis of MIL-101(Cr). The MIL-101(Cr) material doped with praseodymium was subjected to calcination at 400 °C for 4 h in the presence of air. The resulting product, denoted as M-xPrCr, represents praseodymium-doped Cr_2_O_3_, where x/100 corresponds to the molar ratio of Pr/Cr atoms.

#### 3.2.3. Synthesis of P-Cr and P-xPrCr

For comparative purposes, P-Cr and P-xPrCr were synthesized using a conventional precipitation technique. Taking the example of P-30PrCr, a solution was prepared by dissolving 4 g of Cr(NO_3_)_3_·9H_2_O and 1.31 g of Pr(NO_3_)_3_·6H_2_O in 50 mL of deionized water. Subsequently, under vigorous stirring for 2 h, a slow addition of 100 mL of 1 M NaOH solution took place. The resulting precipitate was collected through centrifugation and subjected to three rounds of water washing. Finally, the precipitate underwent drying at a temperature of 80 °C for a duration of 12 h, followed by calcination at an air atmosphere with temperatures maintained at 400 °C for 4 h. The praseodymium-doped Cr_2_O_3_ obtained from this process was denoted as P-xPrCr, where x/100 represents the atom molar ratio between Pr and Cr.

### 3.3. Catalyst Characterization

The catalysts synthesized in this study underwent a systematic characterization process involving techniques such as X-ray diffraction (XRD), scanning electron microscopy (SEM), energy dispersive spectroscopy (EDS), N_2_ adsorption/desorption, X-ray photoelectron spectroscopy (XPS), H_2_ temperature-programmed reduction (H_2_-TPR), and temperature-programmed desorption of ammonia (NH_3_-TPD). The detailed procedures and parameters for these tests can be found in the Supplementary Materials.

### 3.4. Catalytic Test

The performance of catalytic combustion for 1,2-dichloroethane was evaluated in a fixed-bed reactor consisting of a quartz tube with an inner diameter of 4 mm under standard atmospheric pressure. In each experiment, 100 mg of catalysts (40–60 mesh) were immobilized using quartz wool, resulting in a gas hourly space velocity (GHSV) of 20,000 mL·g^−1^·h^−1^ and a total flow rate of 33.33 mL·min^−1^. The liquid reactants (1,2-dichloroethane) were introduced into the feed stream by passing dried air through a saturator maintained at a temperature of 6 °C. The feed stream was then diluted with dried air, generating a feeding flow containing 1000 ppm reactant and composed of 21% O_2_/79% N_2_. Flow rates were regulated using online mass flowmeters. Concentrations of 1,2-DCE and chlorinated by-products were determined via analysis on an online gas chromatograph (GC3420A, Beifen Ruili Co., Ltd., Beijing, China) equipped with both flame ionization detector (FID) and electron capture detector (ECD) for quantitative assessment of organic compounds. Furthermore, the outlet CO_2_ underwent reduction by hydrogen within a methanation furnace before being detected by the same chromatograph system. A K-type thermocouple was inserted into the catalyst bed to monitor its temperature during catalysis. Data of catalytic oxidation tests were collected after achieving steady state conditions for at least thirty minutes.

The 1,2-dichloroethane conversion rate (XDCE, %), CO_2_ yield (CO_2_yield, %), and organic byproduct yield (Y_b_, %) were calculated as follows:

(1) $$X_{DCE}=\frac{{\left(DCE\right)}_{in}-{\left(DCE\right)}_{out}}{{\left(DCE\right)}_{in}}\times 100\%$$

(2) $${CO}_{2}yield=\frac{({{CO}_{2})}_{out}}{2\times {\left(DCE\right)}_{in}}\times 100\%$$

(3) $$Y_{b}=X_{DCE}-{CO}_{2}yield$$

The reaction rate of 1,2- dichloroethane was calculated as follows:

(4) $$r=\frac{Q\times X_{DCE}}{m}$$

where r is the reaction rate (mol·g^−1^·s^−1^), Q is the molar flow of 1,2-dichloroethane (mol·s^−1^), m is the mass of catalyst (g).

Activation energy was calculated via the antiderivative of Arrhenius equation:

(5) $$lnr=lnA-\frac{E_{a}}{RT}$$

where $E_{a}$ is activation energy (kJ·mol^−1^), T is reaction temperature (K), A is a preexponential factor, and R is the molar gas constant (8.314 J·mol^−1^·K^−1^).

The turnover frequency (TOF) was obtained by the following equation:

(6) $$TOF=\frac{Q\times X_{DCE}}{n_{Cr}}$$

where $n_{Cr}$ is the amount of Cr element (mol) in the catalyst.

The stability test for 1,2-dichloroethane was performed at 290 °C for a 120 h continuous test under the same conditions as the catalytic combustion test, unless otherwise indicated.

## 4. Conclusions

In this work, the M-PrCr catalysts were prepared via the in situ pyrolysis of MIL-101(Cr) for 1,2-dichloroethane oxidation. Compared to conventional coprecipitation method, PrCrO_x_ catalysts prepared via pyrolysis strategy exhibited much higher specific surface area and pore volume, which improved the diffusion of 1,2-dichloroethane molecule. In addition, the introduction of Pr-doping effectively increased the number of acidic sites and enhanced the mobility of oxygen species. Consequently, this significantly improved the performance at low temperatures and prolonged the durability of M-PrCr catalysts. M-30PrCr exhibited the highest catalytic performance (T_50_ = 255 °C, T_90_ = 286 °C) and CO_2_ selectivity. Separately, the M-30PrCr catalyst presented exceptional durability against 1,2-DCE and H_2_O during 120 h continues test. In summary, conclusions can be drowned from this study that pyrolysis of MOF strategy and appropriate doping of Pr species can significantly promote the oxidation ability of the catalyst, which provide promising strategy for practical applications in the elimination of 1,2-DCE.

## Figures and Tables

**Table 1:** Structural properties and catalytic performance of synthesized catalysts.

Samples	S_BET_^a^ (m^2^/g)	**Pore Volume ^b^ (cm^3^/g)**	Average Pore Size ^c^ (nm)	T_50_^d^ (°C)	T_90_^d^ (°C)	Cr Content ^e^ (%)	Pr Content ^e^ (%)
M-Cr	135.24	0.36	0.86	320	>400	-	-
M-20PrCr	634.42	0.37	0.71	302	312	16.24	83.53
M-30PrCr	699.31	0.49	0.75	255	286	23.45	76.41
M-40PrCr	648.52	0.44	0.73	271	298	28.29	71.58
P-30PrCr	44.53	0.37	20.24	274	>400	16.96	82.99
P-Cr	57.99	0.20	1.16	371	>400	-	-
P-Pr	19.84	0.21	25.18	>400	>400	-	-

^a^ BET specific surface area. ^b^ Total pore volume estimated at p/p0 = 0.99. ^c^ BJH pore diameter calculated from the absorption branch. ^d^ Temperatures at which 50% and 90% conversions of 1,2-dichloroethane occur. ^e^ The elemental content was quantified using ICP-MS.

**Table 2:** Textural property of prepared catalysts.

Sample	Molar Ratio ^a^		H_2_ Consumption ^b^ (mmol·g^−1^)	Acid Sites		
	O_β_/(O_α_+O_β_)	Cr^6+^/Cr^3+^		P_wa_ ^c^	P_ma_ ^d^	P_sa_ ^e^
M-Cr	0.32	0.97	7.10	95	65	513
P-Cr	0.45	2.16	5.79	51	43	31
P-Pr	- ^f^	-	14.19	130	192	59
M-20PrCr	0.74	1.23	10.61	213	112	585
M-30PrCr	0.81	1.48	27.24	387	122	600
M-40PrCr	0.79	1.41	28.37	363	137	626
P-30PrCr	0.30	4.07	-	-	-	-

^a^ *O*_α_ and *O*_β_ are the surface adsorbed oxygen species and lattice oxygen, respectively; ^b^ H_2_ consumption amount below 700 °C over all samples; Desorption peak areas of NH_3_ absorbed on ^c^ weak acid sites, ^d^ medium acid sites and ^e^ strong acid sites; ^f^ The data that are irrelevant or have not been validated are indicated with a hyphen (-).

## Data Availability

Data will be made available on request.

## Supplementary Materials

The following supporting information can be downloaded at: https://www.mdpi.com/article/10.3390/molecules29143417/s1, Figure S1: TGA analysis of MIL-101(Cr); Figure S2: XRD patterns of samples: (a) MOF-Cr; (b) MOF-30PrCr; (c) M-Cr; (d) M-30PrCr; (e) P-30PrCr; Figure S3: The EDS characterization result for M-30PrCr (total graph); Figure S4: SEM images and EDS mapping results of used catalysts: (a) used M-Cr, (b) used M-30PrCr; Figure S5: The EDS characterization result for (a) used M-Cr and (b) used M-30PrCr (total graph); Figure S6: The XPS Pr 3d spectra for P-30PrCr; Table S1: Surface element distribution of used catalysts.
