# Supplementary material for: Praseodymium-Doped Cr2O3 Prepared by In Situ Pyrolysis of MIL-101(Cr) for Highly Efficient Catalytic Oxidation of 1,2-Dichloroethane
Source: Molecules. 2024 Jul 21;29(14):3417. doi: 10.3390/molecules29143417 (PMC11280410; doi:10.3390/molecules29143417)
Supplement: Supplementary file 1 [file molecules-29-03417-s001.zip › molecules-3032281-Supplementary Materials.pdf]

Supporting Information

Praseodymium-doped Cr_2O_3 prepared by in situ pyrolysis of MIL-101(Cr) for highly efficient catalytic oxidation of 1,2-dichloroethane

Pengfei Zhu, Zhaoxia Hu and Shouwen Chen*

School of Biological and Environmental Engineering, Nanjing University of Science & Technology, Nanjing 210094, China

Keywords: Catalytic oxidation; 1,2-dichloroethane; Praseodymium; In situ pyrolysis; Synergistic effect.

Catalyst Characterization Methods:

The powder X-ray diffraction patterns (XRD) of the samples were collected by a BRUKER D8ADVANCE powder diffractometer with Cu Kα radiation (λ=1.54178 Å) at 40 kV and 40 mA. The patterns were recorded from 5 to 90° at a 10°/min scan speed. Images of scanning electron microscopy (SEM) were obtained using a FEI Quanta 250FEG electron microscope with an acceleration voltage of 3 to 15 kV. The elemental distribution of the samples was detected using Oxford X-Max energy dispersive spectroscopy (EDS). The BET surface area (SBET) and pore volume (Vpore) of the catalysts were determined at 77 K on a Micromeritics ASAP 2020 automatic specific surface analyzer after pretreatment at 200 °C for 2 h under vacuum.

The specific surface area was acquired using the Brunauer-Emmett-Teller equation, and the pore size distribution was measured by the Barrett-Joyner-Halenda (BJH) method. The XPS measurements were carried out on a Thermo ESCALAB 250XI spectrometer equipped with AlKα radiation (1486.6 eV) as the excitation source.

Temperature-programmed reduction by hydrogen (H_2-TPR) was carried out on a conventional apparatus equipped with a thermal conductivity detector (TCD). In each test, the sample (50 mg) was first pretreated at 400 °C for 1 h under a N_2 flow (30 mL/min) and then cooled to room temperature. The test was performed by heating the samples in a 5% H2/Ar (N2) flow (30 mL/min) at a heating rate of 5 °C/min from 50 to 800 °C. The amount of H_2 consumption was calibrated by the complete reduction of a standard 50 mg CuO sample. Temperature-programmed desorption of NH_3 (NH3-TPD) was measured on the same apparatus as H_2-TPR. Before analysis, each sample (100 mg) was pretreated at 400 °C for 1 h with N_2, cooled to 50 °C, saturated with a NH_3 flow (20 mL/min) for 1 h, and then flushed with a N_2 flow (30 mL/min) at the same temperature for 0.5 h to remove the physically adsorbed NH_3. The desorption was performed by heating the samples in a N_2 flow (30 mL/min) from 50 to 800 °C at a heating rate of 5 °C/min. The thermal behavior of MIL-101(Cr) in an air environment was evaluated using a Discovery TGA5500 thermogravimetric analysis (TGA) system with a temperature ramp rate of 5 K/min.

The powder X-ray diffraction patterns (XRD) of samples were recorded on a BRUKER D8ADVANCE powder diffractometer using Cu Kα radiation (λ=1.54178 Å) at 40 kV and 40 mA. The patterns were measured from 5 to 90° at a scanning rate of 10 °/min. Images of scanning electron microscopy (SEM) were obtained via a FEI Quanta 250FEG electron microscope. The elemental distribution of sample was detected using energy dispersive spectroscopy (EDS). The nitrogen adsorption-desorption isotherms were determined at 77 K on a Micromeritics ASAP2020 automatic specific surface analyzer with pretreatment at 200 °C under vacuum for 2 h. The specific surface area was acquired by the Brunauer-Emmett-Teller method, and the pore size distribution was calculated from the desorption branch according to the Barrett-Joyner-Halenda method. The XPS measurements were carried out on a Thermo ESCALAB 250XI spectrometer with AlKα radiation (1486.6 eV) as the excitation source.

Temperature-programmed reduction by hydrogen (H_2-TPR) was carried out in a conventional apparatus equipped with a TCD. The samples (100 mg) were first pretreated for 2 h at 400 °C with a N_2 flow (60 mL/min) before cooled to room temperature. The test was performed by heating the samples in a 5% H_2/N_2 flow (60 mL/min) at a heating rate of 5 °C/min from 50 to 900 °C. The temperature-programmed desorption of NH_3 (NH_3-TPD) was measured on the same apparatus as H_2-TPR. Before measurement, each sample (100 mg) was pretreated at 400 °C for 2 h with N_2, cooled to 50 °C, saturated with 5% NH_3 in Ar. The test was performed by heating the samples in a Ar flow (60 mL/min) at a heating rate of 5 °C/min from 50 to

900 °C. The effluent was quantified by a TCD detector.

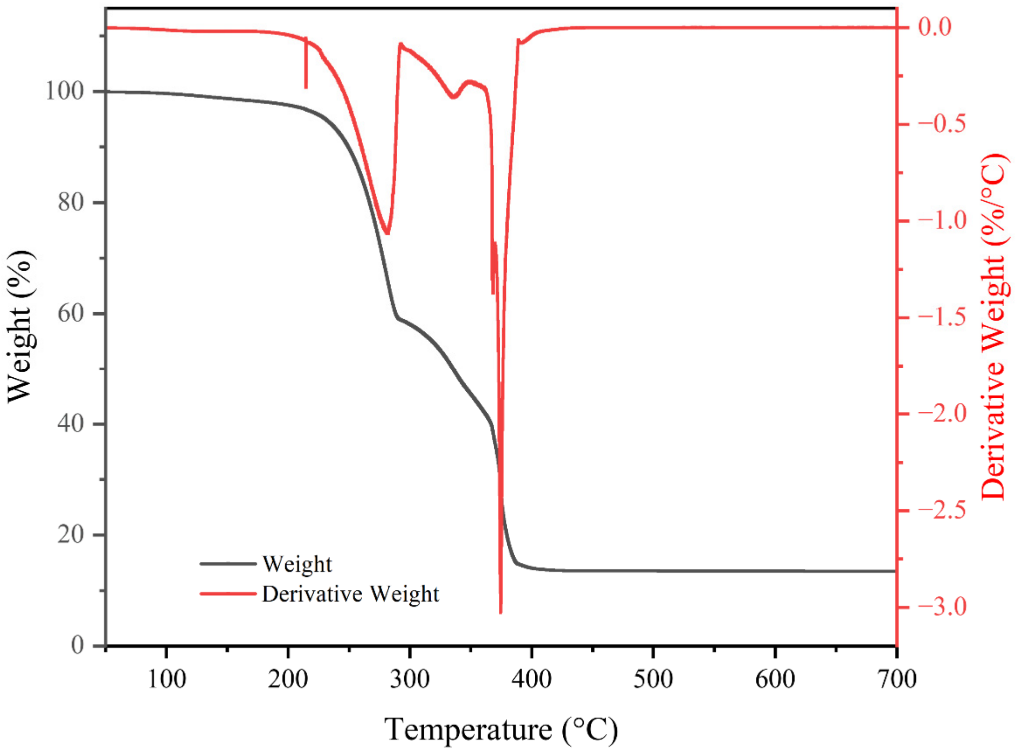

Figure S1. TGA analysis of MIL-101(Cr).

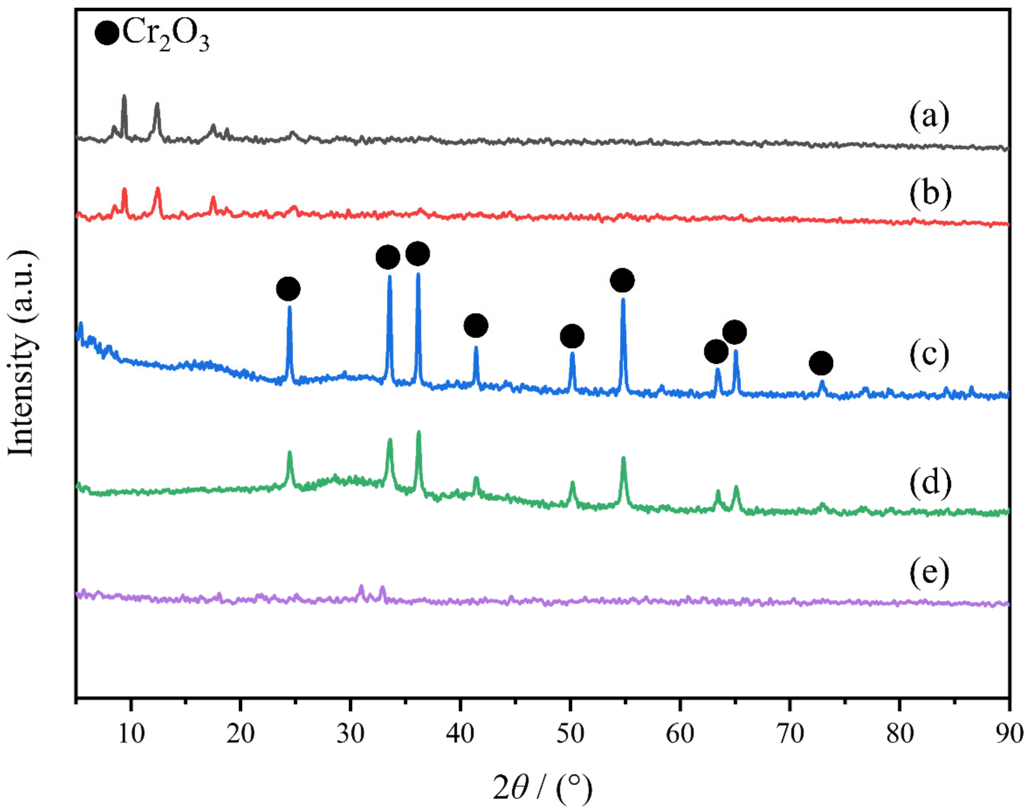

Figure S2. XRD patterns of samples: (a) MOF-Cr; (b) MOF-30PrCr; (c) M-Cr; (d) M-30PrCr; (e) P-30PrCr.

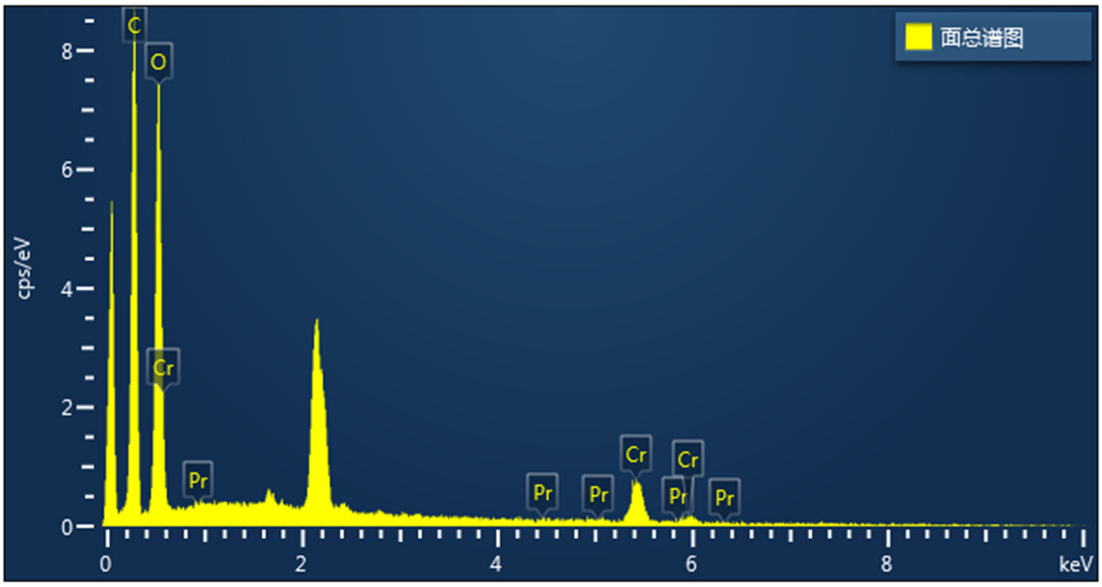

Figure S3. The EDS characterization result for M-30PrCr (total graph).

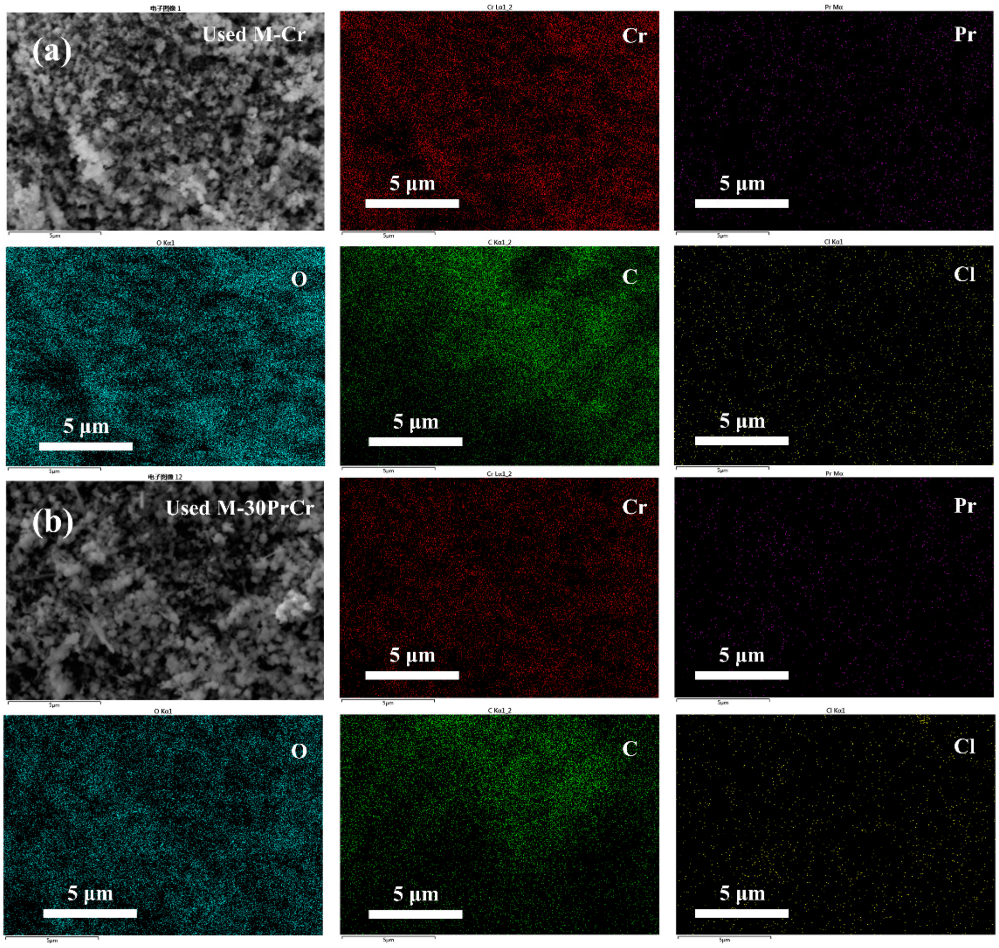

Figure S4. SEM images and EDS mapping results of used catalysts : (a) used M-Cr, (b) used M-30PrCr.

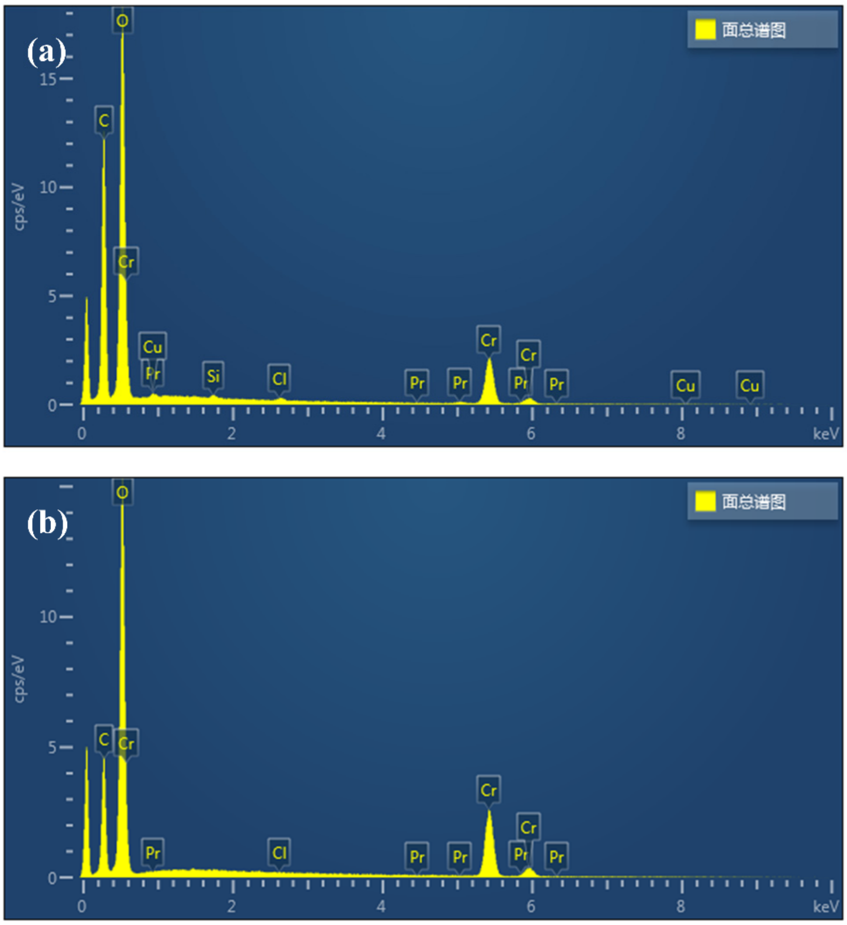

Figure S5. The EDS characterization result for (a) used M-Cr and (b) used M-30PrCr (total graph).

Table S1

Surface element distribution of used catalysts.

Sample	C wt%[a]	Cl wt%	Cr wt%	O wt%
Used M-Cr	21.41	0.73	48.89	24.06
Used m-30PrCr	5.13	0.28	65.49	26.72

[a] The distribution of surface elements on used catalysts is scanned and analyzed by EDS in randomly selected regions.

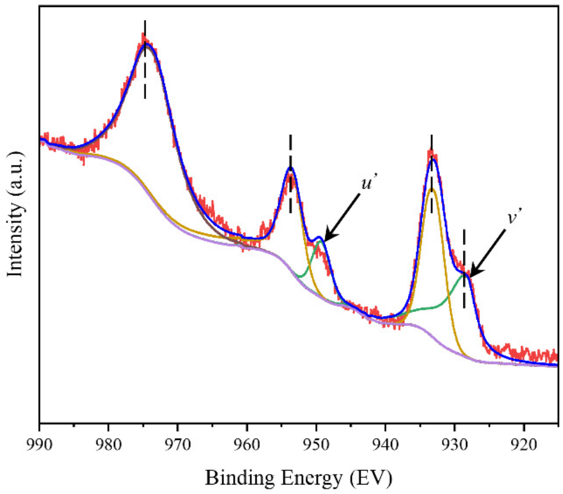

Figure S6. The XPS Pr 3d spectra for P-30PrCr.
